# Effect of peptide length on the conjugation to the gold nanoparticle surface: a molecular dynamic study

**DOI:** 10.1186/s40199-014-0085-2

**Published:** 2015-01-29

**Authors:** Fatemeh Ramezani, Mostafa Habibi, Hashem Rafii-Tabar, Massoud Amanlou

**Affiliations:** Department of Medical Physics and Biomedical Engineering, Shahid Beheshti University of Medical Sciences, Tehran, Iran; Department of Medicinal Chemistry, Faculty of Pharmacy and Pharmaceutical Sciences Research Center, Tehran University of Medical Sciences, Tehran, Iran

**Keywords:** Gold nanoparticles, Interface, Amino acid, Interaction, Molecular dynamic

## Abstract

**Background:**

Gold nanoparticles now command a great deal of attention for medical applications. Despite the importance of nano-bio interfaces, interaction between peptides and proteins with gold surfaces is not still fully understood, especially in a molecular level.

**Methods:**

In the present study computational simulation of adsorption of 20 amino acids, in three forms of mono-amino acid, homo di-peptide and homo tri-peptide, on the gold nanoparticles was performed by Gromacs using OPLSAA force field. The flexibility, stability, and size effect of the peptides on the gold nanoparticles were studied as well as the molecular structure of them.

**Results:**

According to our results, adsorbed homo tri-peptides on the gold surface had more flexibility, more gyration, and the farthest distance from the GNP in comparison with homo di-peptides and mono-amino acids.

**Conclusion:**

Our findings provide new insights into the precise control of interactions between amino acids anchored on the GNPs.

**Electronic supplementary material:**

The online version of this article (doi:10.1186/s40199-014-0085-2) contains supplementary material, which is available to authorized users.

## Background

Both natural processes and biotechnological applications involve the interaction of polypeptides with solid surfaces. For example, the growth of hard tissue is regulated by protein-mineral interactions and the adhesion of organisms to solid supports depends on protein–surface interactions [1]. Gold nanoparticle (GNP) is one of nanomaterials attracted a great deal of attention in medical sciences because of their biocompatibility and ability to conjugate to organic compounds and bio-molecules which make them useful in biochemical sensing and detection [2]. Besides, amino acids are used in bio-functionalization of GNPs as protective layers and they are used for assemble of GNPs [3].

The interface between GNP and amino acids is an important issue in bio-catalysis and biosensor designing [4,5]. Despite the critical importance of nano-bio interfaces, the mechanism of binding of proteins and peptides to surfaces is not still fully understood, especially in a molecular level [1]. While finding actual experimental data for describing and visualizing protein/surface conjugates is difficult, molecular dynamic simulations open a new way to a better understanding of structure and dynamics of even individual atoms [6,7].

In 2007, with the help of molecular dynamic (MD) simulations and COMPASS force field, Zhen Xu et al. showed histidine and histidine containing peptide adsorption behavior on gold surface [5]. In 2010, using GOLP force field, amino acids conformation on the gold surface was founded by Martin Hoefling et al. [7] and also in 2011; they showed the interaction of beta-sheet peptides with a gold surface and found that adsorption occurs in a stepwise mechanism [8].

In 2012, using MD simulation, Lee and Ytreberg explored effect of the GNP conjugation on six different peptides [6]. They showed peptides dynamic and structure depends on the amino acids sequence. In the last paper our group compared 20 natural amino acids interaction with gold nanoparticle by molecular dynamic [9]. Next, we continue to understand the impact of peptide lengths on their adsorption, their flexibility and stability while interacting with GNPs from the molecular level point of view. We compared homo mono, di and tri- amino acids adsorption on the gold nanoparticle surface. Tri-peptides serve as model systems for understanding the so-called random-coil state of peptides and proteins [10].

## Methods

**Figure 1:**
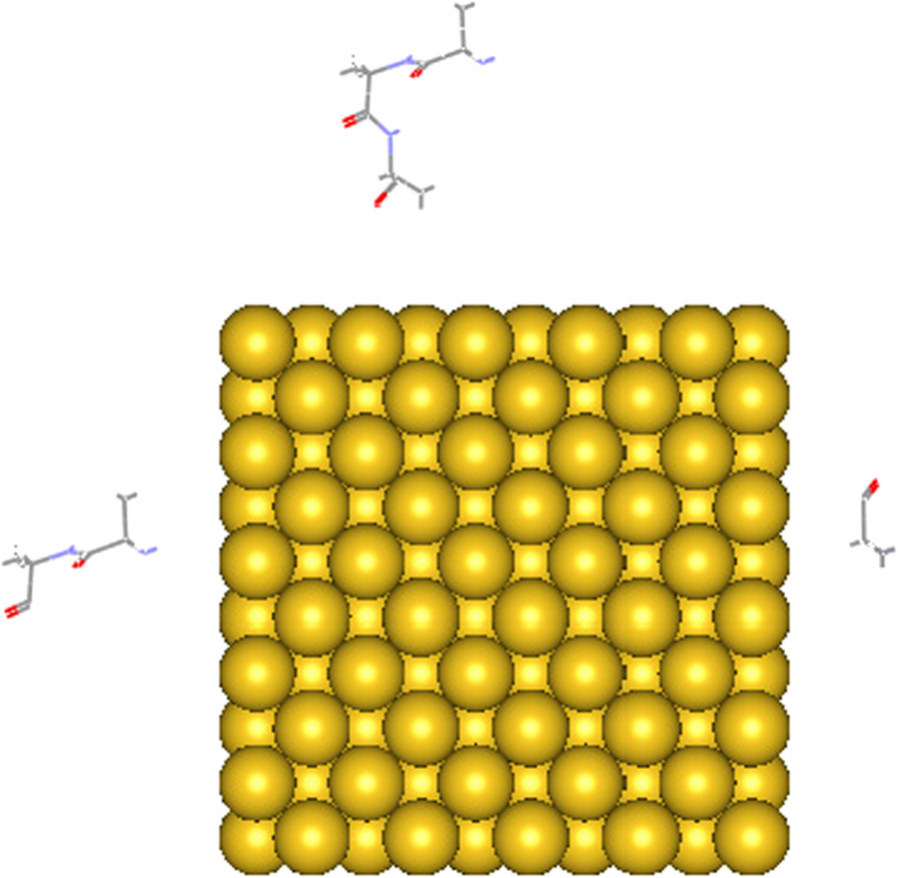
**A sample system was simulated.** This figure shows initial system containing Ala, Ala-Ala and Ala-Ala-Ala before adsorption on the gold nanoparticle.

20 different systems were used for this study (Table 1). Each system contains one kind of amino acid in mono, homo di-, and tri-peptides forms (Figure 1):

**Table 1:** **Amino acid sequences were considered for simulation in this study**

1. Ala, Ala-Ala, Ala-Ala-Ala	2. Val, Val-Val, Val-Val-Val	3. Asp, Asp-Asp, Asp-Asp-Asp
4. Gly, Gly-Gly, Gly-Gly-Gly	5. Ser, Ser-Ser, Ser-Ser-Ser	6. Asn, Asn-Asn, Asn-Asn-Asn
7. Thr, Thr-Thr, Thr-Thr-Thr	8. Pro, Pro-Pro, Pro-Pro-Pro	9. Lys, Lys-Lys, Lys-Lys-Lys
10. Gln, Gln-Gln, Gln-Gln-Gln	11. His, His-His, His-His-His	12. Trp, Trp-Trp, Trp-Trp-Trp
13. Arg, Arg-Arg, Arg-Arg-Arg	14. Phe, Phe-Phe, Phe-Phe-Phe	15. Cys, Cys-Cys, Cys-Cys-Cys
16. Leu, Leu-Leu, Leu-Leu-Leu	17. Tyr, Tyr-Tyr, Tyr-Tyr-Tyr	18. Ile, Ile-Ile, Ile-Ile-Ile
19. Glu, Glu-Glu, Glu-Glu-Glu	20. Met, Met-Met, Met-Met-Met

3 × 3 × 3 nm^3^ cubic shape GNP (111) was built of 2048 atoms by Hyperchem 8.0.3 software. GNP kept its cubic shape after 10 ns simulation time. In a previous study by our group, the effect of the size of nanoparticle was examined and it was found that the cubic nanoparticle size makes no difference on the interaction of amino acids [9]. So in this study GNP size was considered 3 nm.

Peptides and GNP were solvated in a 50 Å^3^ length cubic water box. The distance between the peptides and the box edge was 9 Å. Then each system was ionized with 0.15 mol/l ion concentration using a mixture of Na^+^ and Cl^¯^ ions such that each system had zero net charge. All simulation conditions were set, (normal saline, pH = 7.4, Temperature = 27°C), and kept constant during the simulation.

All simulations were performed in TIP3P water model [11,12]. During the simulation, periodic boundary conditions were applied in all three dimensions. All simulations were performed using Gromacs 4.6.3 software package [13] and OPLSAA force field [14,15]. Despite the perfect force fields such as Sutton-Chen are used for simulation of metals but it was not appropriate for investigating the interaction of metal with peptides. Among several force fields for peptide-gold interactions that have been reported in the literature (e.g. [16-18]) we used OPLSAA force field and gold atom parameters were taken from Hendrik Heinz et al. where r_0_ = 2.951 Å and ε_0_ = 5.29 kcal/mol [19]. As can be find in Hendrik Heinz results, these parameters can be implemented in the OPLS-AA force field and applied to the simulation of GNPs and their interfaces with water, biopolymers, organic, and inorganic molecules. Using these parameters, we can consider all gold atoms in a dynamic mode [19]. This LJ models for fcc metals can be implemented in force fields such as AMBER, CHARMM, COMPASS, CVFF, OPLS-AA, PCFF, and applied to the simulation of metals and their interfaces with water, biopolymers, organic molecules, and inorganic components. This LJ models for fcc metals are typically an order of magnitude more accurate than previous LJ parameters due to the physical interpretation of the quantities r_0_ and ε_0_ in terms of the metal density and the surface tension of the {111} crystal face under standard conditions [19]. During the simulation, center of mass position is fixed.

Discovery Studio 2.1 [20] was used for the preparation of snapshots. Minimization was performed for 100 ps and followed by a 1.0 ns of dynamic simulation in the NPT ensemble and an 8.0 ns dynamic simulation in the NVT ensemble at T = 300 °K. A Nose’-Hoover thermostat was utilized for simulation processes to enforce the desired temperatures [21]. The LINKS algorithm was implemented to allow 8 × 10^−4^ ps time step [22]. The long-range electrostatic interactions were accounted for using the Ewald method with a real-space cut-off of 12 Å. Vander Waals interactions were cut off at 12 Å with a switching function between 10 and 12 Å.

## Results and discussion

### Root mean square deviation (RMSD)

Root mean square deviation (RMSD) (Eq. (1)) showed the calculated system equilibrations as follows [23]:

1 $$ RMSD\left({t}_1,{t}_2\right)={\left[\frac{1}{N}{\displaystyle \sum_{i=1}^N{m}_i\parallel {r}_i\left({t}_1\right)-{r}_i\left({t}_2\right)\parallel}\right]}^{\frac{1}{2}} $$

Where N is the number of target molecules; and ri (t) is the position of molecule i at time t. The analysis of the RMSD graph of alpha carbons during the adsorption of amino acids on the GNP showed that all systems were stable after 2 ns (Additional file 1: Figure S1). This indicated that systems remained stable during the simulation. In Ser and Tyr, the hydroxyl group of side chain and in His and Pro, the ring of side chains constantly fluctuated on the surface of GNP which caused RMSD value increased related to the other amino acids. Only His homo di-peptide placed farther than its homo tri-peptides.

In the last paper published by our group we showed mono amino acids conformations after adsorption on the GNP surface [9]. Our results for Met, Cys, Pro, Phe, Gly and Leu were matched with the experimental results reported by Podstawka et al. In the current work we found di and tri- amino acids conformation after adsorption on the gold surface. These results and comparison with conformation of mono-amino acids were shown in Additional file 2. Our results for Met, Cys, Pro, Phe, Gly di-peptide interactions with gold atoms are also consistent with the experimental results reported by Podstawka et al. [24]. According to the experimental results Met-Met and Cys-Cys interaction with GNP is through sulphur atom that is near to our results. According to the Additional file 2, Gly-Gly adsorption was through –COOH group that is accordance with experimental results. In our simulation results and Podstawka experimental results Phe-Phe, aromatic rings had a main role in adsorption on the gold surface.

In all amino acids except Pro, interacting group with GNP in homo mono-, di-, and tri-peptides didn’t change. Pro interaction in mono-amino acid and homo di-peptide is through N atom. But according to our result by increasing the length of prolin to homo tri-peptide, spatial limitation of left-handed 3(1)-helix conformation of tri-prolin didn’t allow the N atoms to come near the GNPs surface.

### Flexibility of peptides after adsorption on the GNP

Atomic fluctuations were studied using the root-mean square fluctuations (RMSF) analysis [19]. Additional file 2 indicates the last frame of adsorption of peptides on the GNP and the RMSF per residue of homo mono-, di-, and tri-peptides. Almost in all systems, fluctuations of homo tri- peptides were more than homo di-peptides and fluctuations of homo di-peptides were more pronounced than mono-amino acids. Therefore it can be concluded that increasing the peptide length increases the fluctuation. The exception is homo tri-peptide of Arg, Asp and Cys that has lower fluctuation than its homo di-peptide and mono-amino acid. The comparison of the 20 systems revealed that homo tri-peptides with aromatic rings which include Tyr, Phe, Trp have more and Pro has the lowest fluctuations than the other amino acids. Rigidity of the poly-proline reduces the degrees of freedom [25]. In outcome, increasing the aromatic rings in the structure of peptides increases the fluctuation of peptides on the GNP surface.

### Compactness of peptides

In order to have a rough measure of the compactness of a structure, we calculated the radius of gyration as follows (Eq. (2)):

2 $$ {R}_g={\left(\frac{{\displaystyle \sum i\parallel {r}_i{\parallel}^2\;{m}_i}}{{\displaystyle \sum i\kern0.24em {m}_i}}\right)}^{\frac{1}{2}} $$

Where m_i_ is the mass of atom i; and r_i_ is the position of atom i about the centre of mass of the molecule.

Additional file 3 displays the time evolution of radius of gyration (Rg) for homo mono-, di-, and tri-peptides. According to the Rg trajectory and the comparison between the 20 systems, amino acids were divided into three groups. The first group includes Trp, Phe, Tyr, Arg, and Lys which have the most compactness. The second group encompasses Asp, Glu, Met, Leu, His, and Gln. The third, finally, includes Val, Ala, Gly, Asn, Thr, Ser, Pro, Ile, and Cys that have the lowest compactness than two other groups. Among all the amino acids, Arg had the most compactness which may be due to its more linear structure compared to other homo tri-peptides.

### Distance of peptides from the GNP

Calculating and comparing peptides center of mass distances from the center of mass of GNP showed homo tri-peptides that have more flexibility on the GNP, fall farthest away from the nanoparticles. Most of homo di-peptides were also farther than mono-amino acids than the GNP (Additional file 1: Figure S2).

## Conclusions

Amino acids are used for, both in functionalizing of GNPs and in cross-linking of amino acid capped GNPs [26]. Because of the importance of studying the interface between amino acids and GNP, in this study, 20 common amino acid interactions with GNP were performed by a computational simulation. In this paper, we have considered a square-shaped gold nanoparticle. Certainly the interaction of amino acids with surface and the defects on the GNP are different, but the aim of this study was to evaluate the effect of the peptide length from one to three amino acids on their binding to the GNP surface.

Lee and Ytreberg showed presence of GNP alter both the peptide structures and dynamics, and that the magnitude of the effect depends on the peptide sequence [6]. Conjugated peptides typically have decreased conformational flexibility, and the amount of decrease depends on the amino acid sequence. In this study we showed that GNP effects in addition to sequencing, it also depends on the length of the peptides. Homo tri-peptides that were adsorbed on the GNP surface have more flexibility, further radius of gyration, and fall in the farthest distance from the GNP, compared with homo mono and di-peptides.

Aromatic rings in amino acids made them more fluctuating on the GNP surface. These results can be used in amino acids usage as a linker on the GNPs.

These findings improve nanofabrication methods for immobilization of amino acids and proteins on the GNP at molecular precision to control both lateral and perpendicular orientations of peptides, the size, and the kind of amino acids on the GNP.

## Additional files

Additional file 1:
**Due to the large number of figures in the manuscript, RMSD of amino acids and their distance from the gold surface has been shown in Additional file** **1** **.**
Additional file 2:
**Amino acids last snapshot and RMSF.** This figure indicates peptides last frame snapshots (left column), root mean square fluctuations (RMSF) per atom about the time-averaged structure (right column) for mono-amino acids (black line), homo di-peptides (green line) and homo tri-peptides (red line). Additional file 3:
**Amino acids radius of gyration.** Graphs indicate Rg-time of mono-amino acids (black line), dipeptides (green line) and tripeptides (red line) on the GNP. In the images can be seen in all amino acids, with increasing the peptide lengths, they were farther away from the gold surface.
