# Supplementary material for: Effect of peptide length on the conjugation to the gold nanoparticle surface: a molecular dynamic study
Source: Daru. 2015 Jan 29;23(1):9. doi: 10.1186/s40199-014-0085-2 (PMC4312466; doi:10.1186/s40199-014-0085-2)
Supplement: Additional file 1: — Due to the large number of figures in the manuscript, RMSD of amino acids and their distance from the gold surface has been shown in Additional file 1 . [file 40199_2014_85_MOESM1_ESM.pdf]

Supporting information

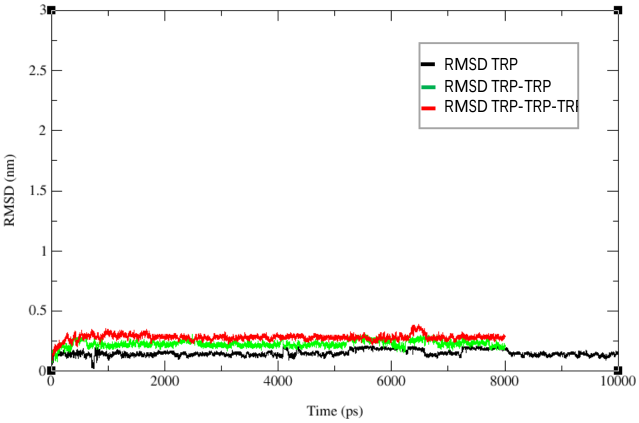

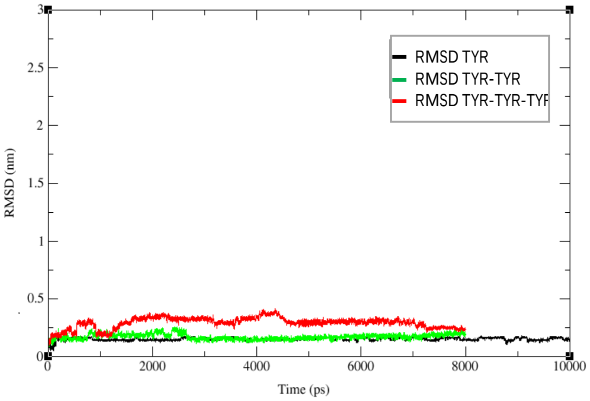

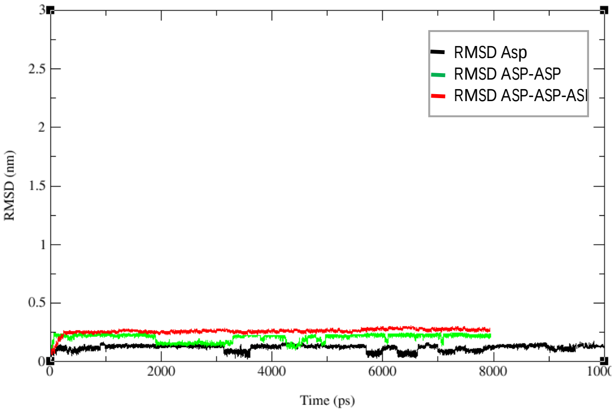

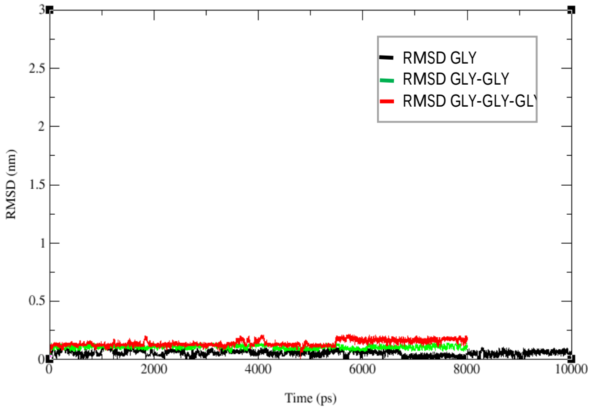

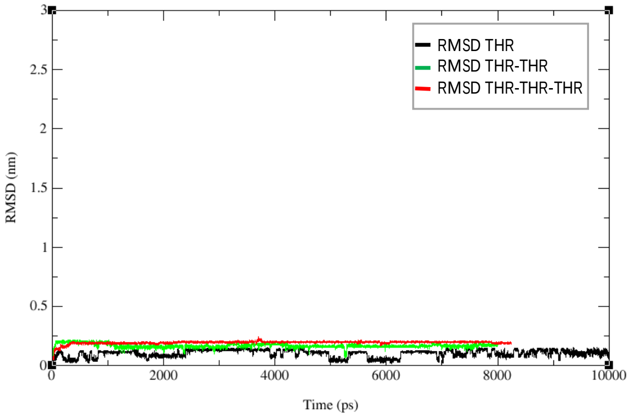

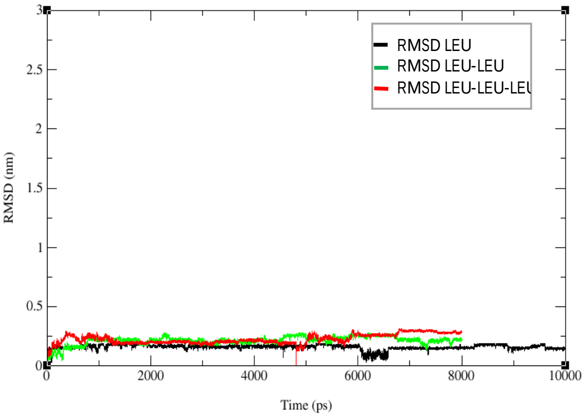

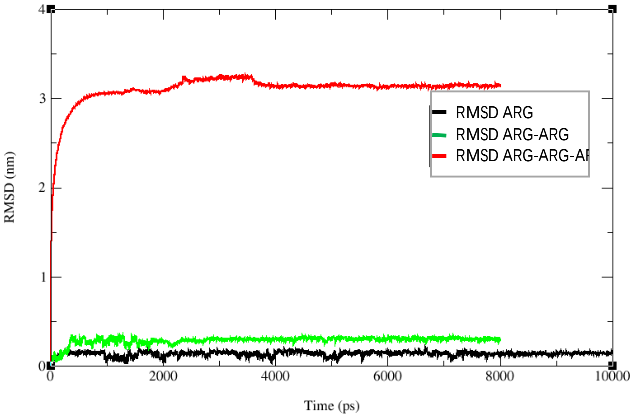

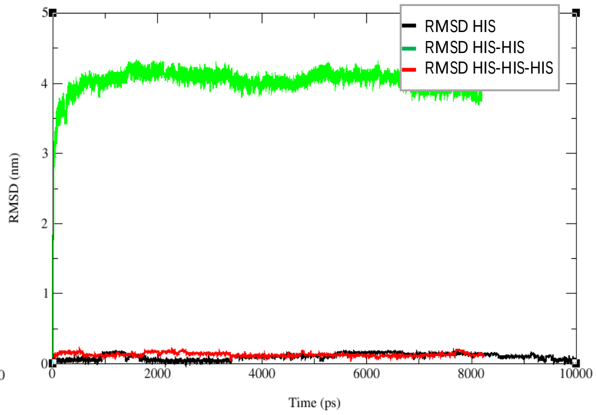

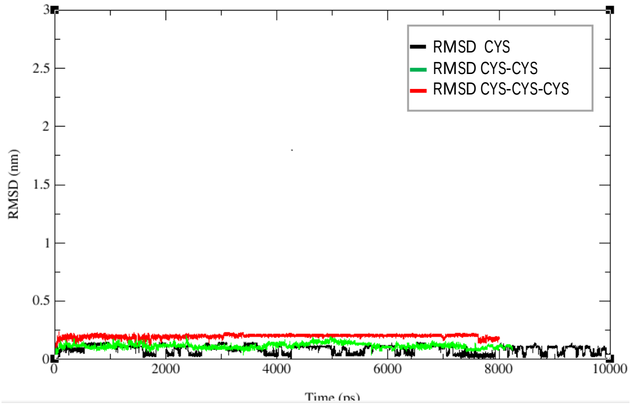
RMSD CYS
RMSD CYS-CYS
RMSD CYS-CYS-CYS
RMSD (nm)
Time (ps)

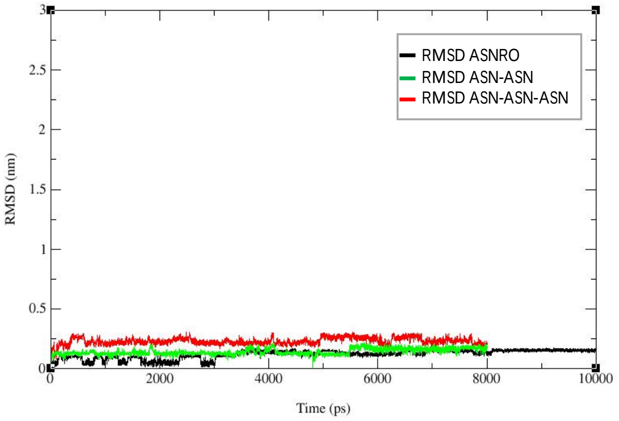
RMSD ASNRO
RMSD ASN-ASN
RMSD ASN-ASN-ASN
RMSD (nm)
Time (ps)

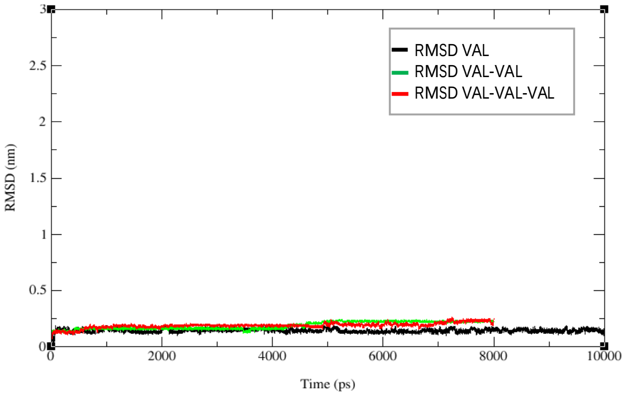
RMSD VAL
RMSD VAL-VAL
RMSD VAL-VAL-VAL
RMSD (nm)
Time (ps)

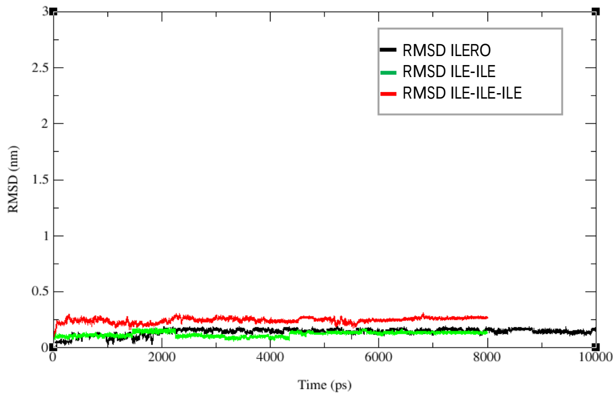
RMSD ILERO
RMSD ILE-ILE
RMSD ILE-ILE-ILE
RMSD (nm)
Time (ps)

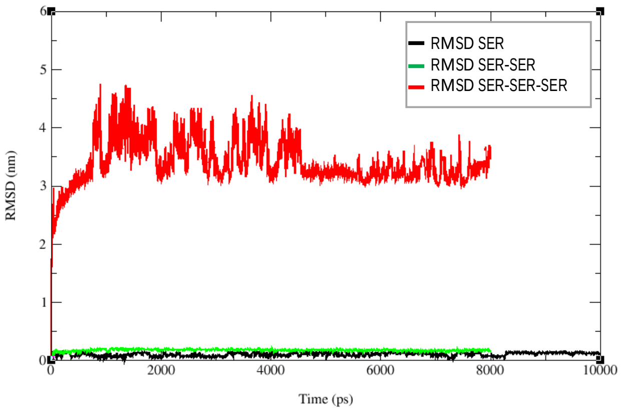
RMSD SER
RMSD SER-SER
RMSD SER-SER-SER
RMSD (nm)
Time (ps)

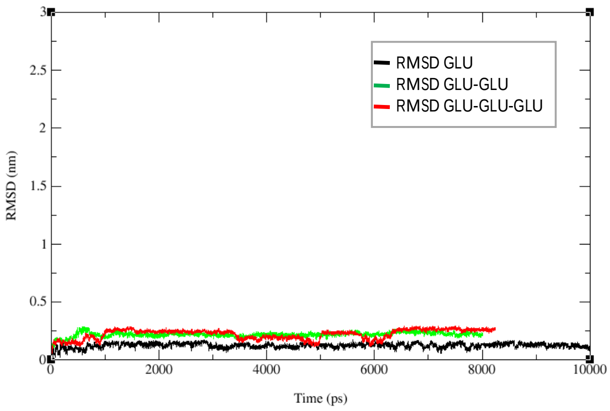
RMSD GLU
RMSD GLU-GLU
RMSD GLU-GLU-GLU
RMSD (nm)
Time (ps)

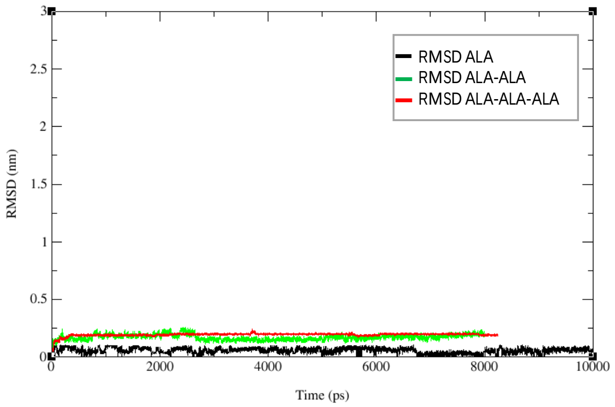
RMSD ALA
RMSD ALA-ALA
RMSD ALA-ALA-ALA
RMSD (nm)
Time (ps)

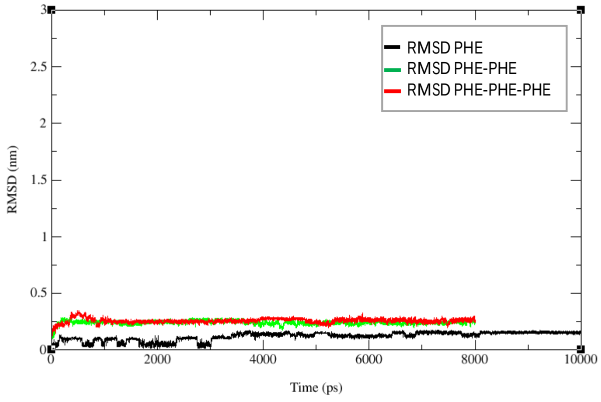
RMSD PHE
RMSD PHE-PHE
RMSD PHE-PHE-PHE
RMSD (nm)
Time (ps)

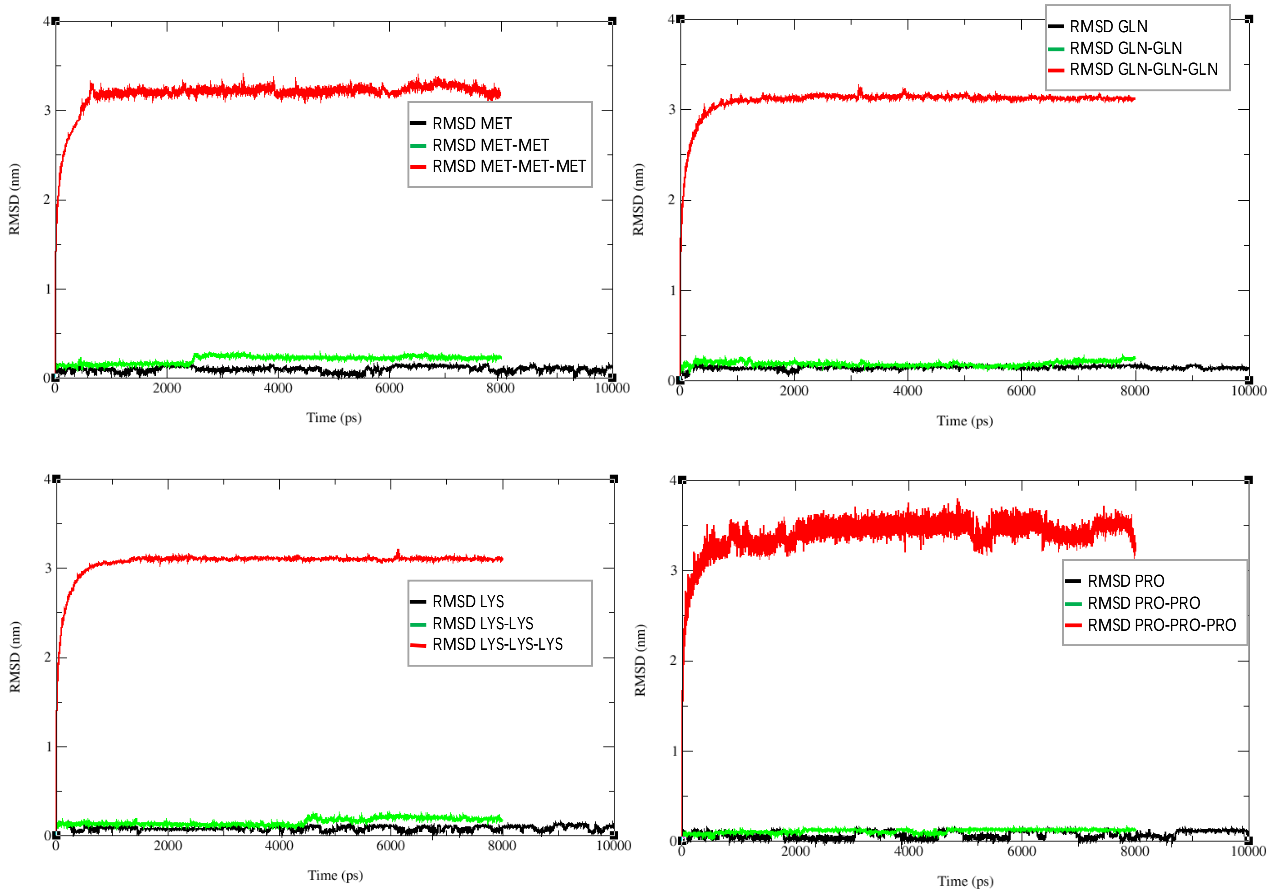

Figure1. This figure shows RMSDs of peptides. Amino acids are black, di-peptides are shown green and homotripeptides are shown by red line. Respectively from up-left to right are Trp,Tyr, Asp, Gly, Thr, Leu, Arg, His, Cys, Asn, Val, Ile, Ser, Glu, Ala,Phe, Met, Gln, Lys, Pro.

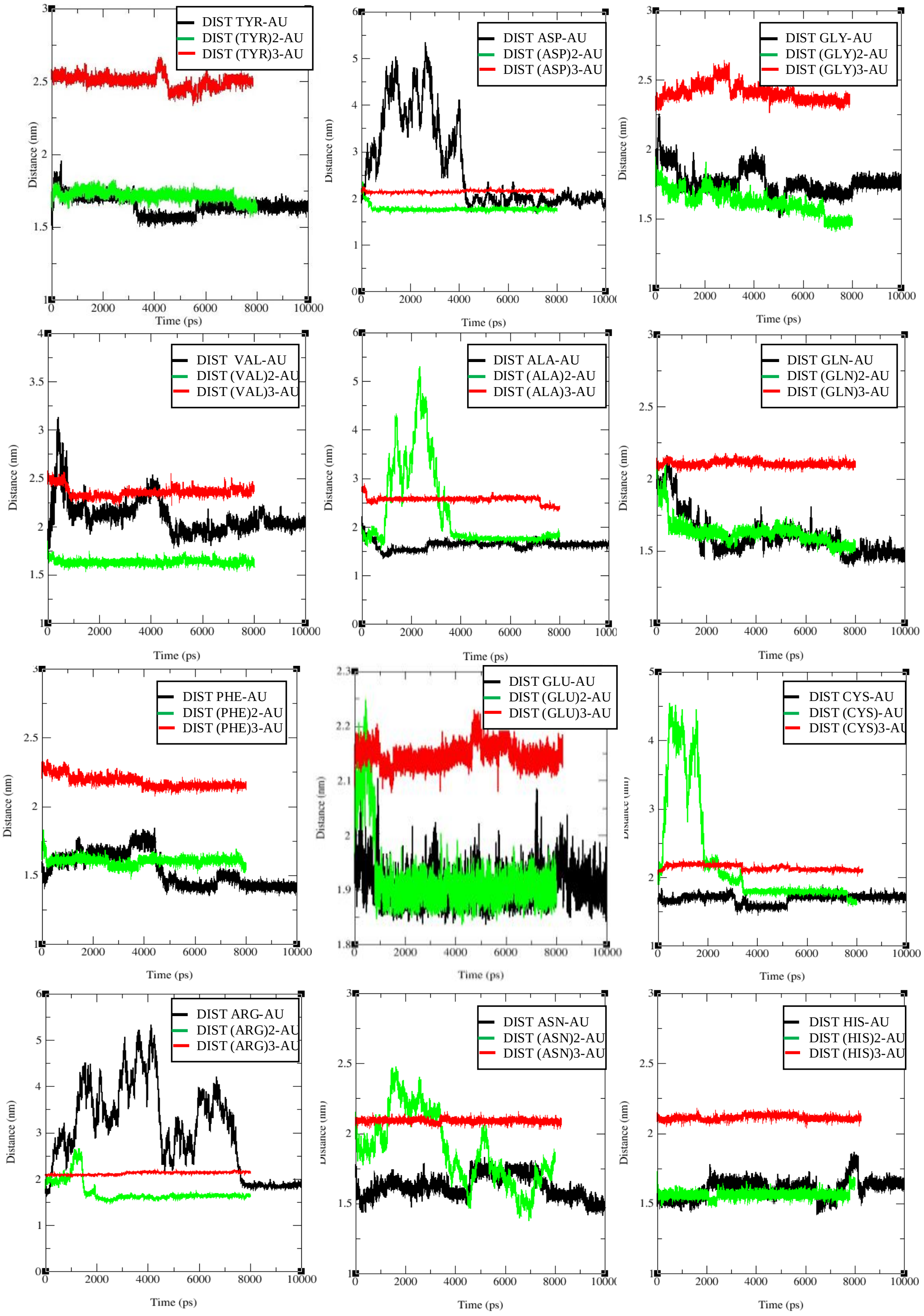

DIST TYR-AU
DIST (TYR)2-AU
DIST (TYR)3-AU
DIST ASP-AU
DIST (ASP)2-AU
DIST (ASP)3-AU
DIST GLY-AU
DIST (GLY)2-AU
DIST (GLY)3-AU
DIST VAL-AU
DIST (VAL)2-AU
DIST (VAL)3-AU
DIST ALA-AU
DIST (ALA)2-AU
DIST (ALA)3-AU
DIST GLN-AU
DIST (GLN)2-AU
DIST (GLN)3-AU
DIST PHE-AU
DIST (PHE)2-AU
DIST (PHE)3-AU
DIST GLU-AU
DIST (GLU)2-AU
DIST (GLU)3-AU
DIST CYS-AU
DIST (CYS)-AU
DIST (CYS)3-AU
DIST ARG-AU
DIST (ARG)2-AU
DIST (ARG)3-AU
DIST ASN-AU
DIST (ASN)2-AU
DIST (ASN)3-AU
DIST HIS-AU
DIST (HIS)2-AU
DIST (HIS)3-AU
Distance (nm)
Time (ps)

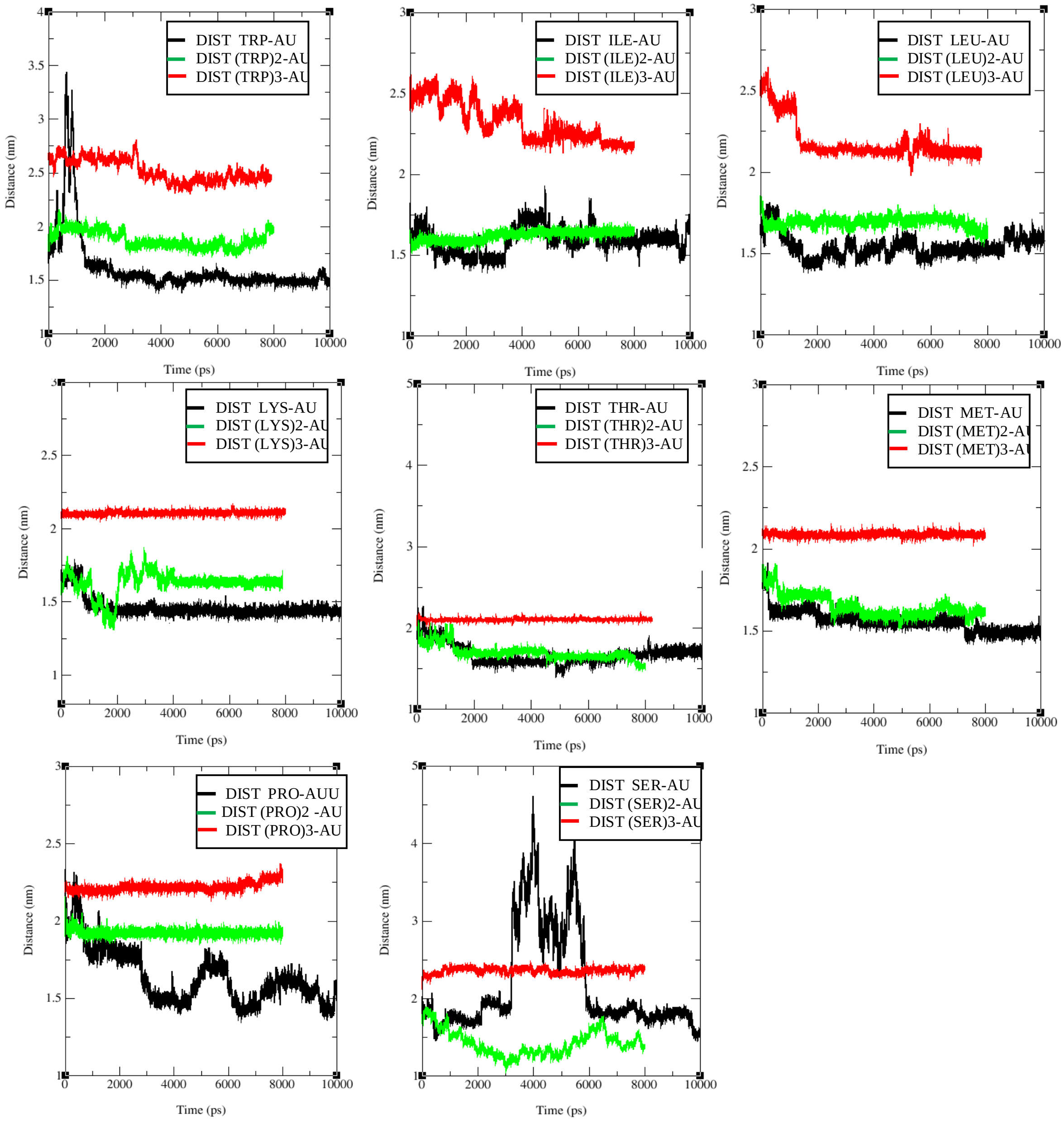

Figure 2. Distance of mono-amino acids (black), dipeptides (green), tripeptides (red) from GNP.
