# Supplementary figures and images for: Effect of peptide length on the conjugation to the gold nanoparticle surface: a molecular dynamic study
Source: Daru. 2015 Jan 29;23(1):9. doi: 10.1186/s40199-014-0085-2 (PMC4312466; doi:10.1186/s40199-014-0085-2)

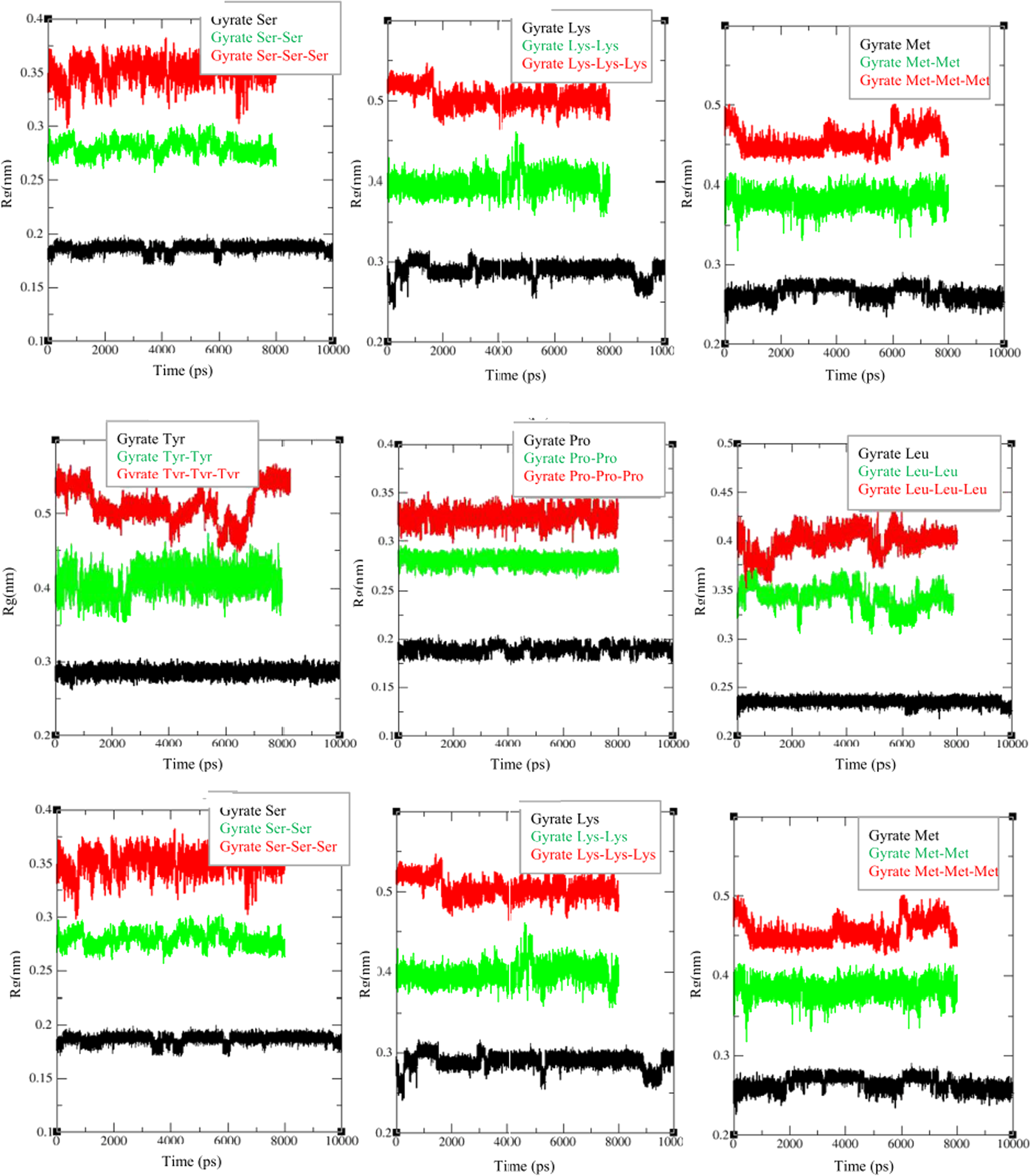

Supplement: Additional file 2: — Amino acids last snapshot and RMSF. This figure indicates peptides last frame snapshots (left column), root mean square fluctuations (RMSF) per atom about the time-averaged structure (right column) for mono-amino acids (black line), homo di-peptides (green line) and homo tri-peptides (red line). [file 40199_2014_85_MOESM2_ESM.zip › 40199_2014_85_add3a.tif]

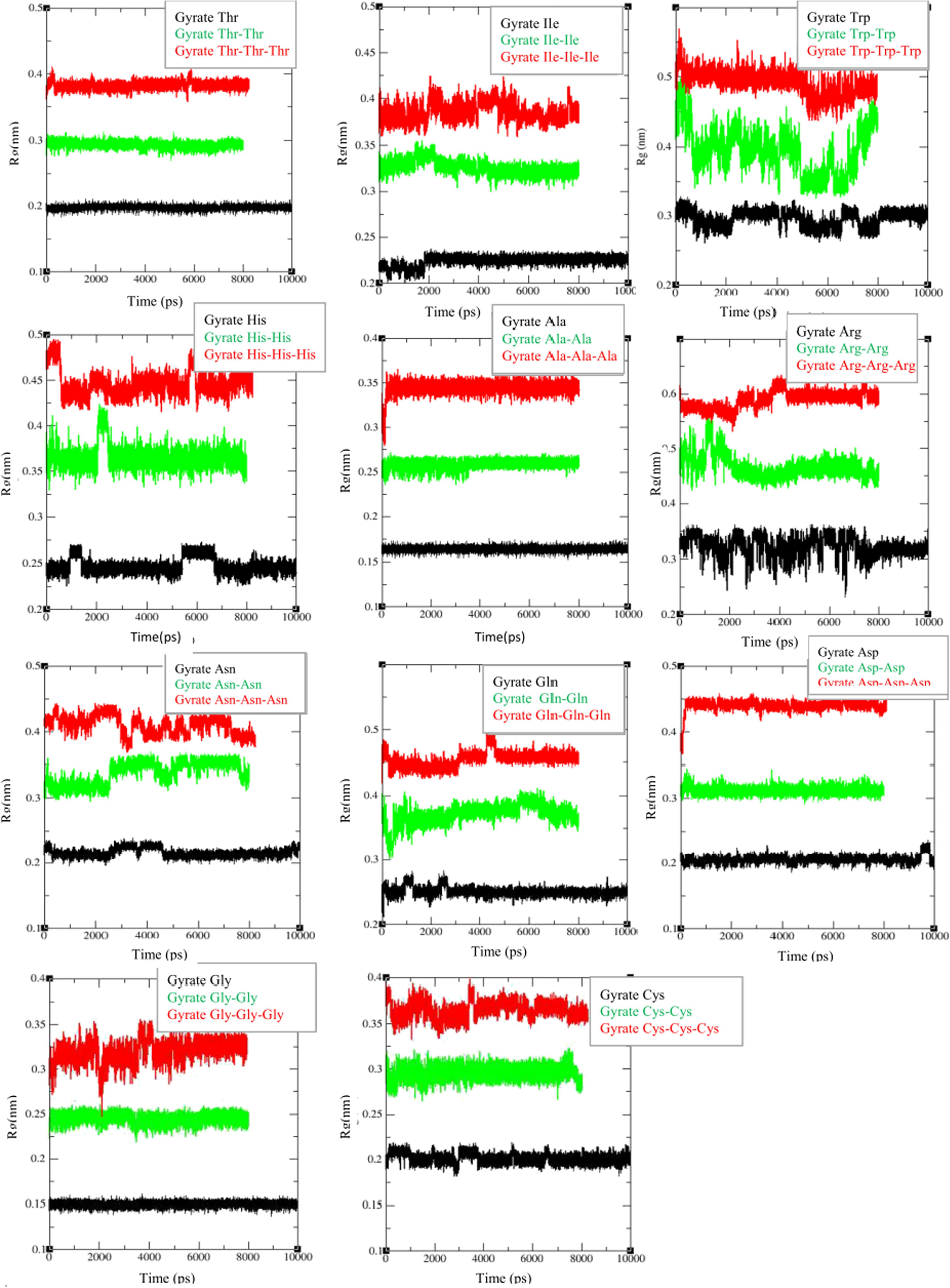

Supplement: Additional file 2: — Amino acids last snapshot and RMSF. This figure indicates peptides last frame snapshots (left column), root mean square fluctuations (RMSF) per atom about the time-averaged structure (right column) for mono-amino acids (black line), homo di-peptides (green line) and homo tri-peptides (red line). [file 40199_2014_85_MOESM2_ESM.zip › 40199_2014_85_add3b.tif]
